# A Charge‐Coupled Phototransistor Enabling Synchronous Dynamic and Static Image Detection

**DOI:** 10.1002/adma.202417675

**Published:** 2025-04-14

**Authors:** Shun Feng, Ruyue Han, Chi Liu, Dayu Jia, Guoteng Zhang, Xi Zhu, Bo Li, Yun Sun, Chuang Li, Yuping Gao, Tonglei Cheng, Zheng Han, Hui‐Ming Cheng, Dong‐Ming Sun

**Affiliations:** ^1^ Shenyang National Laboratory for Materials Science Institute of Metal Research Chinese Academy of Sciences 72 Wenhua Road Shenyang 110016 China; ^2^ School of Materials Science and Engineering University of Science and Technology of China 72 Wenhua Road Shenyang 110016 China; ^3^ School of Information Institution Liaoning University 66 Chongshan Road Shenyang 110036 China; ^4^ School of Physical Science and Technology Shanghai Tech University 393 Huaxiazhong Road Shanghai 200031 China; ^5^ State Key Laboratory of Synthetical Automation for Process Industries College of Information Science and Engineering Northeastern University 11 Wenhua Road Shenyang 110819 China; ^6^ State Key Laboratory of Quantum Optics and Quantum Optics Devices Institute of Opto‐Electronics Shanxi University 92 Wucheng Road Taiyuan 030006 China; ^7^ Faculty of Materials Science and Engineering/Institute of Technology for Carbon Neutrality Shenzhen Institute of Advanced Technology Chinese Academy of Sciences 1068 Xueyuan Avenue Shenzhen 518055 China

**Keywords:** charge‐coupling effect, dynamic detection, machine vision, phototransistor, static detection

## Abstract

Emerging machine vision applications require efficient detection of both dynamic events and static grayscale information within visual scenes. Current dynamic vision and active pixel sensors (DAVIS) technology integrates event‐driven vision sensors and active pixel sensors within single pixels. However, the complex multi‐component pixel architecture, typically requiring 15–50 transistors, limits integration density, increases power consumption, and complicates clock synchronization. Here, a charge‐coupled phototransistor is presented that uses dual photosensitive capacitors to provide gate voltage to a single transistor channel, enabling simultaneous capture of dynamic and static information, surpassing existing DAVIS technology. Under illumination, both top and bottom gates generate photogenerated electrons through a charge‐coupling effect; electrons in the top gate are blocked by a thick dielectric layer, producing a stable current change for static grayscale detection, while electrons in the bottom gate tunnel through a thin dielectric layer, creating transient current spikes for dynamic event detection. This device demonstrates a dynamic range of 120 dB and a response time of 15 µs, comparable to traditional DAVIS pixels, while significantly reducing power consumption to 10 pW and overcoming clock synchronization issues. This charge‐coupled phototransistor paves the way for the development of high‐performance, low‐power, and highly integrated machine vision technology.

## Graphical abstract

A charge‐coupled phototransistor is presented that uses dual photosensitive capacitors to provide gate voltage to a single transistor channel, enabling simultaneous capture of dynamic and static information, surpassing existing DAVIS technology. This charge‐coupled phototransistor paves the way for the development of high‐performance, low‐power, and highly integrated machine vision technology.

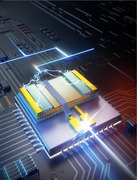

## 1 Introduction

**Figure 1:**
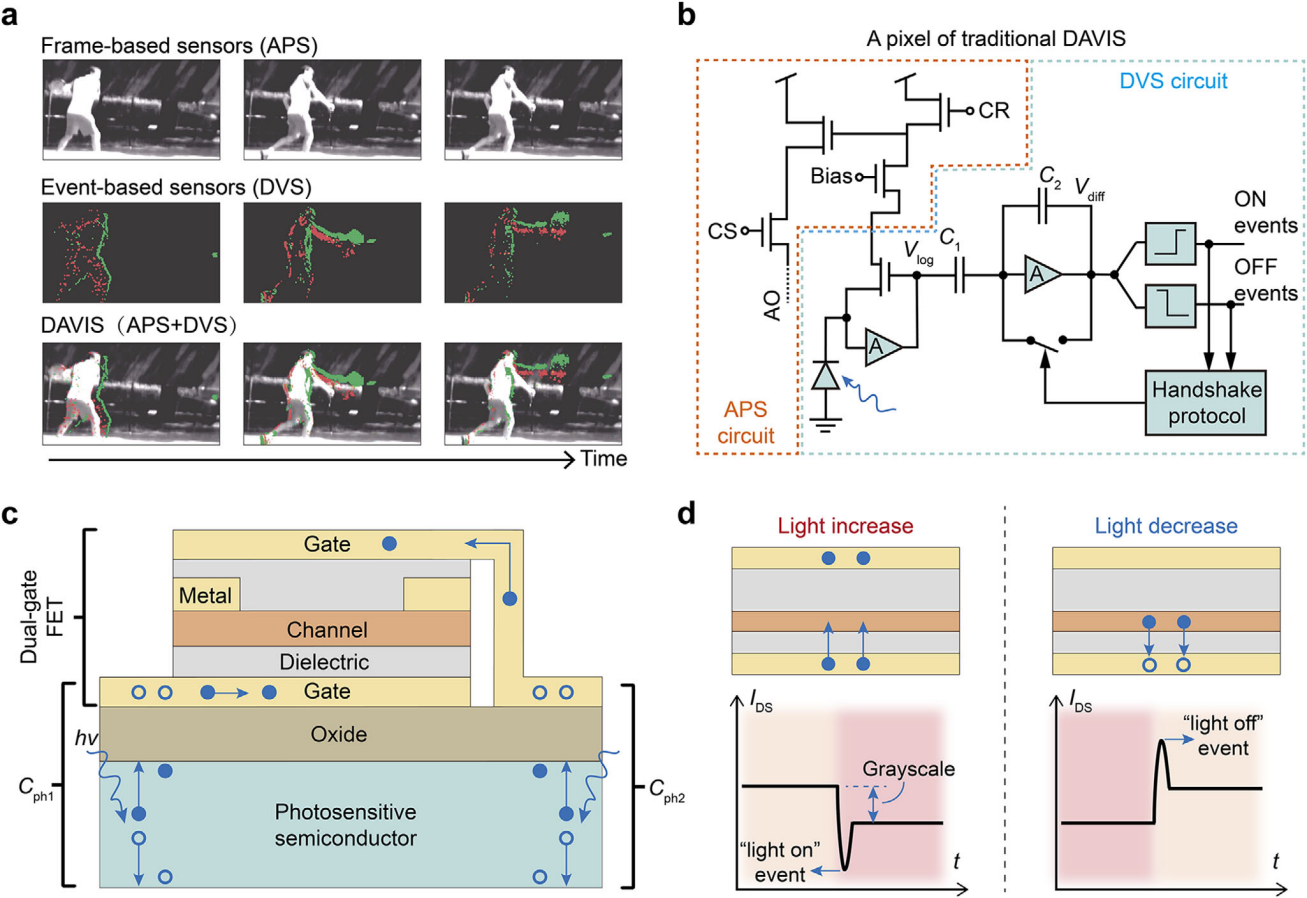
Charge‐coupled phototransistor enabling synchronous detection of events and grayscale information. a) Comparison between APS, DVS, and DAVIS. APS record all pixels at a fixed frame rate, capturing both dynamic motion and static background, while DVS capture only changes in light intensity with high temporal resolution. DAVIS combine the features of APS and DVS within a single pixel, enabling both event and grayscale detection, but complicating clock synchronization. b) Pixel circuit of traditional DAVIS, which requires 15–50 transistors. c) Design of the charge‐coupled phototransistor, utilizing dual photosensitive capacitors (*C*_ph_) to provide the gate voltage for the channel (n‐type). d) Dual dielectric of different thicknesses enable a single transistor to detect both events (light intensity changes) and grayscale (absolute light intensity).

The advancement of machine vision technologies is pivotal for enhancing the performance and reliability of autonomous driving and robotics systems,^[^ ^1^, ^2^ ^]^ which require efficient hardware for detecting both dynamic events and static grayscale information within visual scenes.^[^ ^3^, ^4^, ^5^, ^6^ ^]^ Traditional active pixel sensors (APS), while proficient in capturing grayscale images by transmitting absolute light intensity at a fixed frame rate, fall short in real‐time response (**Figure** **1**a).^[^ ^7^, ^8^ ^]^ In contrast, event‐driven dynamic vision sensors (DVS) transmit information only when detecting changes in light intensity,^[^ ^9^, ^10^, ^11^, ^12^, ^13^, ^14^, ^15^, ^16^, ^17^, ^18^, ^19^ ^]^ excelling in temporal resolution and data bandwidth efficiency but lacking in grayscale information,^[^ ^20^, ^21^, ^22^ ^]^ which limits their performance in applications requiring high spatial resolution and detailed capture (Figure 1a).

To bridge the gap between APS and DVS, the dynamic and active pixel vision sensors (DAVIS) integrate both into a single pixel.^[^ ^23^, ^24^, ^25^, ^26^, ^27^ ^]^ The DAVIS pixel detects light intensity changes and outputs asynchronous event data through the DVS component, while the APS component provides synchronous exposure for grayscale information (Figure 1a). Despite the substantial potential of DAVIS technology, its complex multi‐component pixel structure presents significant challenges. On one hand, DAVIS pixels typically require 15–50 transistors, constraining integration and increasing power consumption (Figure 1b). For instance, the 240 × 180 DAVIS reported by Brandli et al., with each pixel consisting of 47 transistors, 3 capacitors, and 1 photodiode, resulting in a static power consumption of 116 nW per pixel.^[^ ^23^ ^]^ On the other hand, achieving precise time synchronization between high temporal resolution DVS and frame‐based APS poses notable difficulties (Figure 1b),^[^ ^9^, ^10^ ^]^ necessitating advanced synchronization techniques to ensure accurate data fusion and prevent timing discrepancies.^[^ ^20^, ^21^ ^]^ These challenges underscore the need for a more integrated and efficient solution.

In this work, we propose a charge‐coupled phototransistor that utilizes dual photosensitive capacitors to provide gate voltage for the channel, enabling a single transistor device to capture both dynamic events and static grayscale information concurrently (Figure 1c). Using the charge‐coupling effect, photogenerated electrons in the top gate are blocked by a thicker dielectric layer, inducing stable current change for static grayscale detection (Figure 1d). Meanwhile, photogenerated electrons in the bottom gate tunnel through a thinner dielectric layer, resulting in transient current spikes for dynamic event detection (Figure 1d). Our device achieves a dynamic range of 120 dB and a delay time of 15 µs, comparable to traditional DAVIS pixels, while significantly reducing the power consumption to 10 pW per pixel. Crucially, this design enables precise control over the timing synchronization between event and grayscale detection, effectively addressing the clock synchronization issues inherent in conventional DAVIS designs.

## 2 Results

### 2.1 Device Design and Characteristics

**Figure 2:**
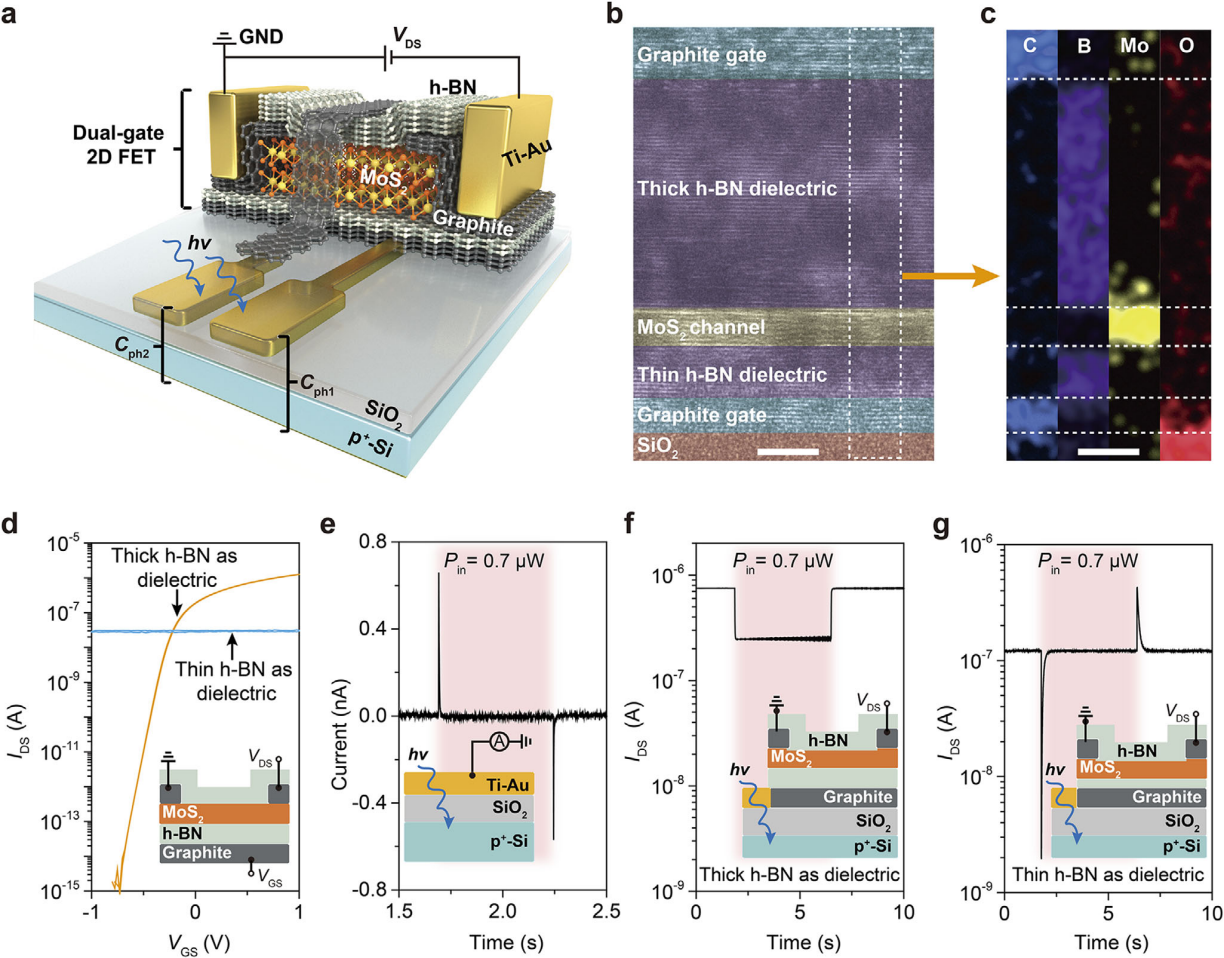
Structure and characteristics of charge‐coupled MoS_2_ phototransistors. a) Schematic of the charge‐coupled phototransistor composed of a dual‐gate MoS_2_ FET integrated with two Ti‐Au/SiO_2_/p^+^‐Si photosensitive capacitors (*C*_Ph_), utilizing thin and thick h‐BN as the bottom and top dielectric layers, respectively. Graphite layers serve as the contact and gate electrodes for improved conductivity. b,c) False‐color transmission electron microscope (TEM) image and elemental maps of C, N, Mo, O of the cross section of the dual‐gate 2D FET, showing high‐quality interfaces of the heterojunctions (scale bar: 5 nm), the thickness of MoS_2_ is about 5 nm. d) Electronic behaviors of the back‐gate 2D FET depends on the thickness of h‐BN dielectric layer, *V*_DS_ = 0.1 V. Insert: structure of the back‐gate 2D FET. e) The optoelectronic behavior of the Ti‐Au/SiO_2_/p^+^‐Si photosensitive capacitor. Insert: structure of the photosensitive capacitor. f,g) Typical photoelectric characteristics of a 2D FET connected with single photosensitive capacitor. Depending on the thickness of h‐BN dielectric layer, the device operates as a photodetetctor or an event‐driven pixel. Insert: structure of the transistors.

To demonstrate the charge‐coupled phototransistor, we designed a dual‐gate 2D field effect transistor (FET) integrated with two silicon‐based photosensitive capacitors (*C*_ph_) (see the Experimental Section and Figure S1, Supporting Information). In the device, graphite served as the contact and gate electrodes, molybdenum disulfide (MoS_2_) as the channel, a thin hexagonal boron nitride (h‐BN) as the bottom dielectric layer, and a thick h‐BN as the top dielectric layer (**Figure** **2**a; Figure S2, Supporting Information). The cross‐section of the dual‐gate 2D FET reveals a van der Waals heterojunction of distinct materials, free of gaps, defects, and contamination (Figure 2b,c). The performance of the 2D FET is significantly influenced by the thickness of the h‐BN dielectric layer. When a thick h‐BN of 10 nm was utilized, the FET operated at a small gate bias and exhibited negligible hysteresis (≈5 mV), an exceptionally high on‐to‐off current ratio (≈10^10^), a very low subthreshold swing (≈65 mV dec^−1^), and good Ohmic contacts (Figure 2d; Figure S3, Supporting Information). In contrast, when a thin h‐BN layer of 3 nm was used, no significant gate control effect was observed, primarily due to the direct tunneling effect of charges through the thin h‐BN layer (Figure 2d).

The photosensitive capacitor features a simple metal/insulator/semiconductor (MIS) structure,^[^ ^28^ ^]^ composed of Ti‐Au/SiO_2_/p^+^‐Si stack. Notably, the thicknesses of Ti and Au are 1 and 3 nm, respectively, making the structure conductive while sufficiently thin for light transmission. Upon light exposure, a positive current spike is measured, indicating that photo‐generated electrons are emitted from the Ti–Au layer. Conversely, when the light is removed, an equivalent number of electrons return to the Ti–Au layer, resulting in a negative current spike (Figure 2e).

By integrating the back‐gate 2D FETs with a single photosensitive capacitor, two distinct photoresponse behaviors are observed depending on the thickness of the h‐BN dielectric layer. When a thick h‐BN layer of 10 nm is used as the dielectric, the device operates as a traditional photodetetctor, showing negative photoconductivity (Figure 2f). In contrast, when a thin h‐BN layer of 3 nm is employed, this device transitions from a photodetetctor to an event‐driven pixel, responding only to the changes in light power. Thus, a negative current spike is generated at the moment of the increase of transient light power (Figure 2g). Reversely, when the light power decreases, positive current spike is produced (Figure 2g). The switching ratio (*I*_Dark_/*I*_Light_) depends on the capacitance ratio between photosensitive capacitor and the gate stack, which can be controlled during device scaling (Figure S4, Supporting Information).

### 2.2 Charge‐Coupling Effect

**Figure 3:**
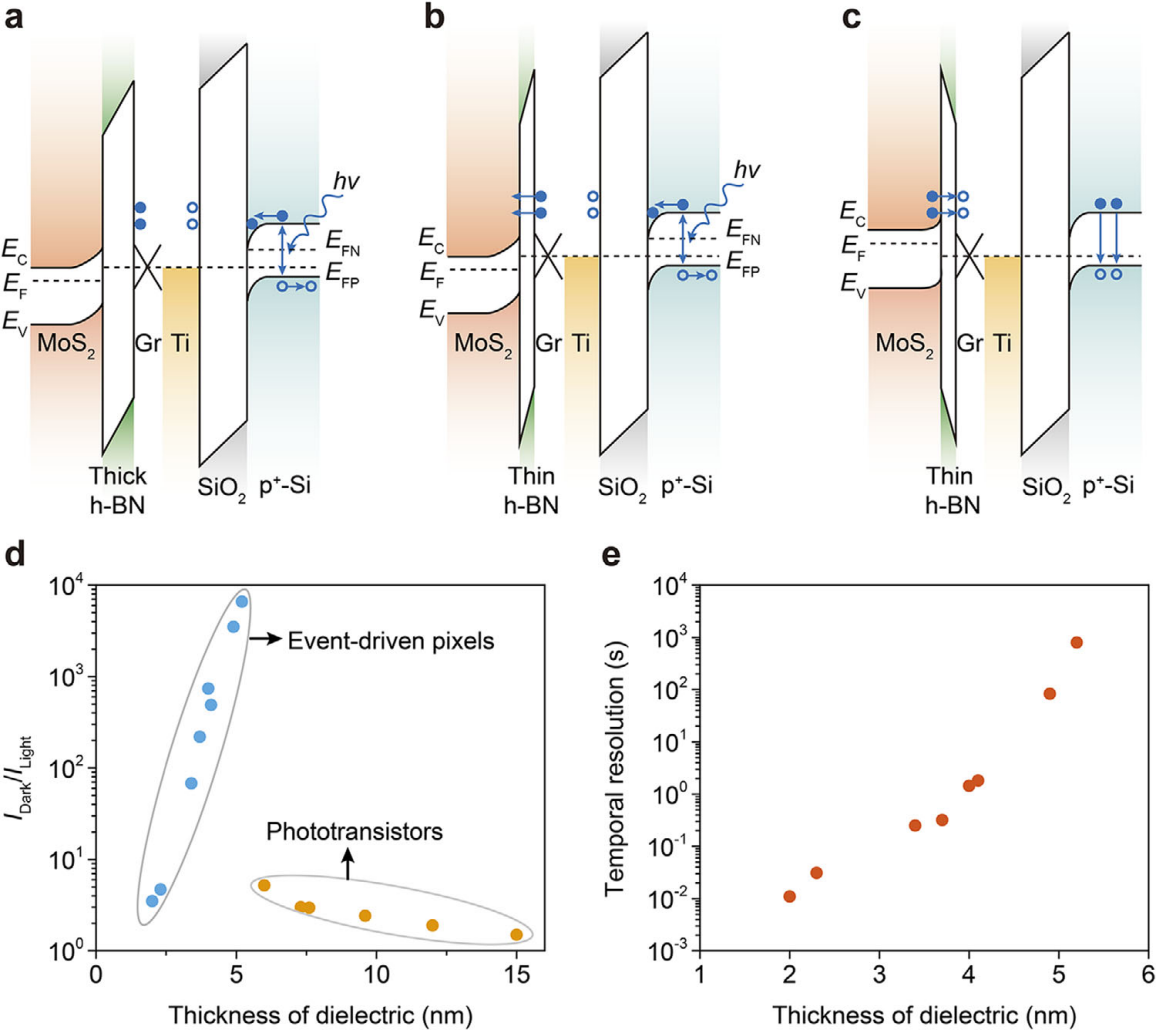
Charge‐coupling effect. a–c) Band diagrams of a back‐gate 2D FETs connected with a single photosensitive capacitor, using a thick or thin b,c) h‐BN as the dielectric layer. *E*_C_ is the conduction band minimum, *E*_F_ is the Femi energy level and *E*_V_ is the valence band maximum. *hv* denotes the incident light; the blue balls and circles indicate negative and positive charges, respectively. d) Photoresponse behavior of transistors as a function of dielectric layer thickness under 516‐nm light with an incident light power (*P*_in_) of 700 nW at *V*_DS_ = 0.1 V. The definition of *I*_Dark_/*I*_Light_ is the ratio of the device currents in dark (*I*_Dark_) and under light (*I*_Light_). For the event‐driven pixles, *I*_Light_ is the peak value of the current spike. e) Temporal resolution of event‐driven pixels as a function of dielectric layer thickness. The temporal resolution refers to the minimum time interval in which the sensor can capture and respond to changes in light intensity.

The disparate photoresponse behaviors observed in transistors with varying h‐BN dielectric layers are attributed to a charge‐coupling effect (Figure 2f,g). As shown in **Figure** **3**a, illumination of the p^+^‐Si results in the excitation of electron–hole pairs. Within the MIS structure of the photosensitive capacitor, a built‐in electric field in the p^+^‐Si facilitates the accumulation of photo‐generated electrons at the SiO_2_/p^+^‐Si interface, inducing positive charges in the metal (Ti–Au) via the charge‐coupling effect. Given the electrically neutral conditions in the interconnected Ti–Au and graphite gate, negative charges (electrons) are driven towards the graphite gate and blocked by the thick h‐BN dielectric, thereby applying a negative gate voltage to the MoS_2_ channel, resulting in a negative photoconductive response (Figure 2e,f). This mechanism, devoid of trap involvement, endows the device with an exceptionally rapid response speed, characterized by a fall time of approximately 10 µs and a rise time of ≈500 µs (Figure S5, Supporting Information).

When the thickness of the h‐BN dielectric layer is reduced, the electrons induced by the charge‐coupling effect are unable to remain in the graphite but instead tunnel through the thin dielectric layer, leading to a negative current spike (Figures 2g and 3b). By removing the light, the electron population at the SiO_2_/p^+^‐Si interface diminishes, thereby reducing the induced positive charge at the metal. This decrease draws electrons from the graphite gate, leaving behind positive charges. These positive charges attract electrons that tunnel back through the thin h‐BN, generating a positive current spike (Figures 2g and 3c).

To validate the proposed mechanism, devices with h‐BN dielectric layers of varying thicknesses (ranging from 2 to 15 nm) were fabricated. It was observed that at an h‐BN thickness of ≈5.5 nm, the device transitions from operating as a photodetector to an event‐driven pixel (Figure 3d; Figure S6, Supporting Information), consistent with the direct tunneling theory.^[^ ^29^ ^]^ When the thickness of the h‐BN dielectric layer is relatively large, electron tunneling is suppressed, and the graphite/ h‐BN/MoS_2_ stack in the phototransistor serves a gate capacitor. As the thickness of the h‐BN layer decreases, the gate control of the device becomes stronger. Therefore, for the same incident light and electron accumulation in the graphite due to the charge coupling effect (Figure 3a), the photogating effect is enhanced leading to a more obvious change of *I*_Light_ and an increased *I*_Dark_/*I*_Light_ (Figure 3d).

In the above case, the electron accumulation in the graphite will repulse subsequent coming electrons by electrical field effect. However, when the h‐BN layer becomes thin enough so that the electron tunneling dominates, the electron repulsion in the graphene weakens, leading to more electrons accumulating in the graphite, which significantly increases the photogating effect and *I*_Dark_/*I*_Light_ (Figure 3d). When the h‐BN thickness continues to decrease, the enhanced tunneling of electrons will decrease the accumulation in the graphite, causing a decrease of the *I*_Dark_/*I*_Light_ (Figure 3d). Additionally, the accumulation and redistribution of electrons in the graphite require a certain relaxation time. As the h‐BN layer becomes thinner, electrons are more likely to tunnel rather than accumulate, resulting in shorter response times for the device (Figure 3e).

### 2.3 Photoelectric Performance of Charge‐Coupled MoS_2_ Phototransistors

A back‐gate 2D FET connected to a single photosensitive capacitor functions solely as a photodetector or an event‐driven pixel. To integrate both photoelectric behaviors into a single transistor, a dual‐gate MoS_2_ FET with two photosensitive capacitors was fabricated, featuring a 10‐nm‐thick top dielectric layer and a 2‐nm‐thick bottom dielectric layer.

**Figure 4:**
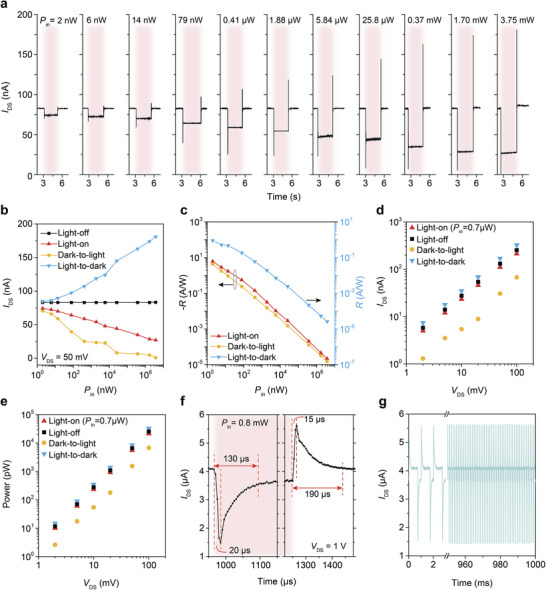
Optoelectronic performance of the charge‐coupled MoS_2_ phototransistor. a) Photoresponse behavior of the transistor at different light power levels (*P*_in_). b) *I*_DS_ of the transistor in four states as a function of *P*_in_ under 516‐nm light at *V*_DS_ = 50 mV, showing a dynamic range (*DR*) of more than 120 dB, which is calculated by *DR* = 20 log (Max *P*_in_/Min *P*_in_). c) Responsivity (*R* = (*I*_Light_‐*I*_Dark_)/*P*_in_)^[^ ^32^ ^]^ of the device for light power and light change detection extracted from (b). d) Photoresponse behavior of the device under different bias voltage using a 516‐nm light with a *P*_in_ of 0.7 µW. e) Power consumption of the device under different bias voltage using a 516‐nm light with a *P*_in_ of 0.7 µW. The power consumption (P) was calculated using the formula: P = *I*_DS_* *V*_DS_​, where *I*_DS_ and *V*_DS_ are the drain current and voltage of the device, respectively​. f,g) Optical switching response of the device under 516‐nm light with *P*_in_ of 0.8 mW and 1000 Hz frequency, exhibiting a 15 µs latency for light‐on and 20 µs for light‐off, with temporal resolutions of 130 and 190 µs, respectively.

Under illumination, electrons in the two graphite gates simultaneously apply negative gate voltage to the MoS_2_ channel layer, enabling the simultaneous detection of events and grayscale information (**Figure** **4**a; Figure S7, Supporting Information). Due to the encapsulation of MoS_2_ by h‐BN and the high‐quality interfaces, the transistor exhibits ultra‐low noise current (Figure 4a,b; Figure S8, Supporting Information),^[^ ^30^ ^]^ allowing accurate detection of both events and grayscale information even with a light power as low as 2 nW (Figure 4a). The detectable light power of this phototransistor ranges from 2 nW to 3.75 mW, showing a dynamic range of more than 120 dB (Figure 4a–c). Due to the transistor itself can detect both events and grayscale, it has extremely low power consumption. The device can operate at a bias voltage as low as 2 mV, with a static power consumption of just 10 pW (Figure 4d,e). Furthermore, due to the trap‐free photogating mechanism, the response speed for detecting both events and grayscale information is intrinsically fast, with a latency of 15 µs and a temporal resolution of 130 µs (Figure 4f). The phototransistor also demonstrates excellent stability and reliability (Figure S9, Supporting Information), with negligible decay in photoresponse behavior after 30000 switching cycles (Figure 4g; Figure S10, Supporting Information). To validate the universality of the device design, we also constructed carbon nanotube‐based charge‐coupled phototransistors (Figure S11, Supporting Information).

**Figure 5:**
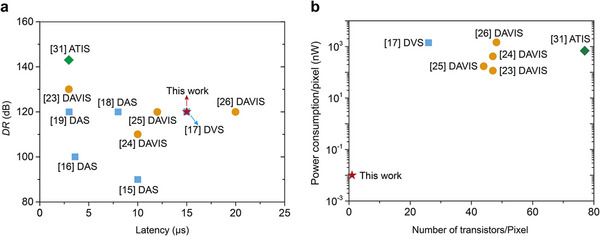
Benchmarks of the charge‐coupled phototransistor against traditional event‐based sensors. a) Comparison of dynamic range (*DR*) and latency, showing comparable performance. b) Significant advantages in power consumption and integration density.

To benchmark our device, we compared traditional multi‐component event‐based sensors including DVS,^[^ ^15^, ^16^, ^17^, ^18^, ^19^ ^]^ DAVIS,^[^ ^23^, ^24^, ^25^, ^26^ ^]^ Asynchronous Time based Image Sensors (ATIS)^[^ ^31^ ^]^ comprehensively. Our charge‐coupled phototransistor demonstrates comparable dynamic range and latency (**Figure** **5**a; Table S1, Supporting Information). At the same time, our device offers significant advantages in terms of power consumption and integration (Figure 5b). Because our charge‐coupled phototransistor consists of only a single transistor and the dual photosensitive capacitors, the overall device size is 5.5 × 7 µm^2^ (Figure S2, Supporting Information), the device is smaller than the pixels of all existing traditional DAVIS (Table S1, Supporting Information). By simultaneously reducing both the area of the photosensitive capacitors and the MIS capacitor of the transistor, our device can be further miniaturized while maintaining excellent performance (Figure S12, Supporting Information). With the future integration of this prototype device into silicon‐based semiconductor processes, we expect to achieve even smaller device sizes, enabling the high‐density integration of photodetector arrays (Figure S13, Supporting Information).

### 2.4 Sensor Arrays

**Figure 6:**
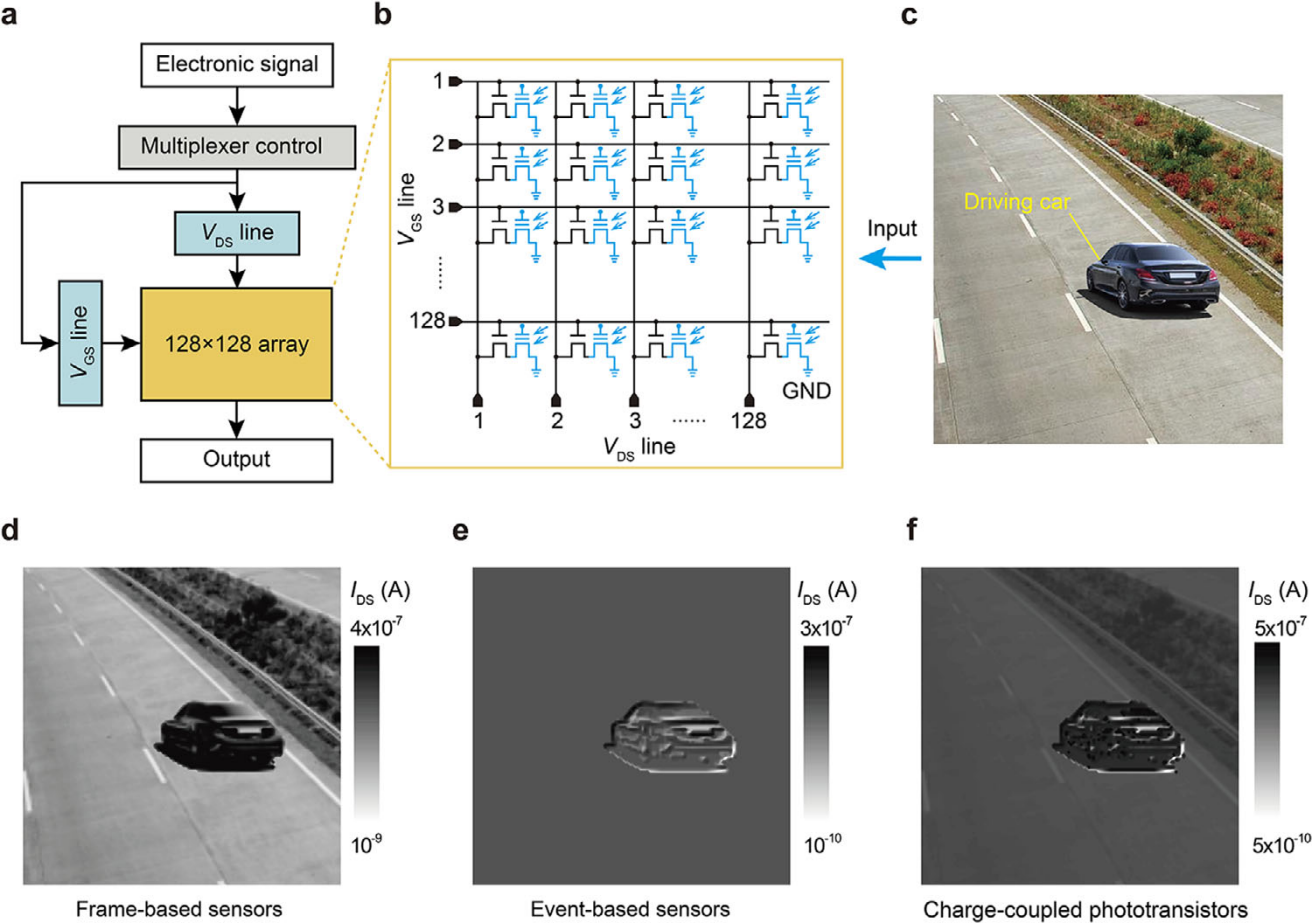
Applications of the charge‐coupled phototransistors in image sensing. a) The structure of the stimulated 128 × 128 image sensors including a multiplexer control, an electronic signal input and output. b) The equivalent circuit diagram of the array. c) A scene containing a driving car and static roads was inputted into the sensors. d–f) Image outputs by the frame‐, event‐ and charge‐coupled phototransistor‐based image sensors.

To demonstrate the potential of the charge‐coupled MoS_2_ phototransistors in image sensors, a 128 × 128 transistor array is designed in a crossbar configuration as an image sensor (**Figure** **6**a), with the equivalent circuit diagram shown in Figure 6b. A pixel contains a charge‐coupled phototransistor (in blue) for light sensing and a field effect transistor (in black) for suppressing crosstalk between pixels. The simulation was carried out using python scripts to model the array's behavior based on our device parameters. The simulation was performed under idealized conditions, assuming perfect pixel isolation and ideal materials, and this simplification is useful for demonstrating the potential of the sensor array. The gray values of the input image were linearly fitted with the power range of detectable light, and were further converted into current values (Figure S14, Supporting Information).

As shown in Figure 6c, a scene containing a driving car and a static road was input to the image sensor based on charge‐coupled transistors (Figure S15, Supporting Information). A traditional camera based on frame‐based sensors conveys absolute light power into current, both the static roads and moving car were recorded (Figure 6d). If the purpose is to determine moving targets and their directions of movement, we must compare multiple images. When the photosensitive components of the camera are event‐based sensors, the parts of the scene where the light power (gray values) changes will be recorded, however, the static roads cannot be detected (Figure 6e). As for the camera composed of charge‐coupled MoS_2_ phototransistors, it can detect both static roads and dynamic car, where the moving target shows more obvious change of current (Figure 6f).

## 3 Conclusion

In conclusion, we have developed a charge‐coupled phototransistor that integrates DAVIS pixel functionality into a single‐transistor device. By utilizing dual photosensitive capacitors to provide gate voltage for the channel, our device achieves a dynamic range of 120 dB and a latency of 15 µs while reducing pixel power consumption to 10 pW. The charge‐coupling effect enables the transistor to detect dynamic events and static grayscale information simultaneously, addressing the clock synchronization challenges of traditional DAVIS. This innovation represents a significant advancement in vision sensors, paving the way for the development of high‐performance, low‐power, and highly integrated machine vision technology.

## 4 Experimental Section

### Device Fabrication

Step 1: material preparation. Graphite, MoS_2_, and h‐BN were exfoliated from their bulk crystals using Scotch tape and were placed on a SiO_2_/p^+^Si substrate. Step 2: heterojunction stacking. h‐BN as the encapsulation material was picked up using a piece of propylene‐carbonate (PPC), followed by the sequential pick up of graphite as the source/drain electrodes, MoS_2_ as the channel, h‐BN as the dielectric, and graphite as the gate electrode. The stack was placed on a surface of a 300‐nm‐thick SiO_2_ layer at 130 °C which had been grown on a heavily p‐doped or n‐doped silicon wafer (0.2–0.02 Ω cm^−1^), followed by heating at 350 °C for 120 min in a vacuum to remove the PPC. Step 3: metal deposition. A polymethyl methacrylate (PMMA) layer (495k MW, A4, MicroChem) was spin‐coated at 2000 rpm on the substrate and baked at 190 °C for 5 min, and another PMMA layer (950k MW, A2, MicroChem) was then spin‐coated at 4000 rpm and baked at 190 °C for 2 min. An undercut structure was created by electron‐beam lithography (EBL) and developing processes. The h‐BN on the graphite source/drain electrodes was then removed using reactive ion etching (RIE) (CHF_3_ with a flux rate of 20 sccm; O_2_ with a flux rate of 4 sccm; pressure, 2.0 Pa; power, 100 W; etching time, 1 min). Metal contacts for the graphite source/drain (Ti/Au: 5/50 nm) electrodes were formed using electron‐beam evaporation and lift‐off processes. Finally, a thin metal pad (Ti/Au: 1/3 nm) for the photosensitive capacitor, the connecting wire of the metal pad and the graphite gate were formed.

### Characterizations

The materials and devices were characterized using an optical microscope (Nikon ECLIPSE LV100ND), an AFM (Bruker Dimension Icon) and an aberration‐corrected TEM (Thermo ScientificTM, Titan Cube Themis G2), with the operating voltage at 300 kV and Super‐X detector system for Energy‐Dispersive X‐ray spectrometry (EDX) mappings. The electrical and photoelectric performance was measured using a semiconductor analyzer (Agilent B1500A), a probe station (Cascade M150) and a laser diode controller (Thorlabs ITC4001, with laser excitations of 516 nm in a dark room at room temperature. The response time was measured using a semiconductor analyzer (Fs Pro, 100 kHz bandwidth) and a laser diode controller (Thorlabs ITC4001).

### Image Sensor Simulations

The three‐mode 128 × 128 image sensors were simulated by python scripts. First, the relationship between the gray values of the input image (0–255) and the detectable light power of the device (0.3 nW–70 µW) was linearly fitted. Then, the relationship between the current (*I*_DS_) and light power of frame‐based sensors, event‐based sensors and charge‐coupled phototransistors can also be abstracted (Figure S14, Supporting Information). A self‐designed scene including cars on both moving and static roads (Figure S15, Supporting Information) was input into the simulated image sensors, which can output current images.

## Conflict of Interest

The authors declare no conflict of interest.

## Supporting information



Supporting Information

## Data Availability

The data that support the findings of this study are available from the corresponding author upon reasonable request.
