# Supplementary material for: A Charge‐Coupled Phototransistor Enabling Synchronous Dynamic and Static Image Detection
Source: Adv Mater. 2025 Apr 14;37(24):2417675. doi: 10.1002/adma.202417675 (PMC12177851; doi:10.1002/adma.202417675)
Supplement: Supplementary file 1 — Supporting Information [file ADMA-37-2417675-s001.docx]

Supporting Information

A charge-coupled phototransistor enabling synchronous dynamic and static image detection

Shun Feng, Ruyue Han, Chi Liu^*^, Dayu Jia, Guoteng Zhang, Xi Zhu, Bo Li, Yun Sun, Chuang Li, Yuping Gao, Tonglei Cheng, Zheng Han, Hui-Ming Cheng & Dong-Ming Sun^*^

**
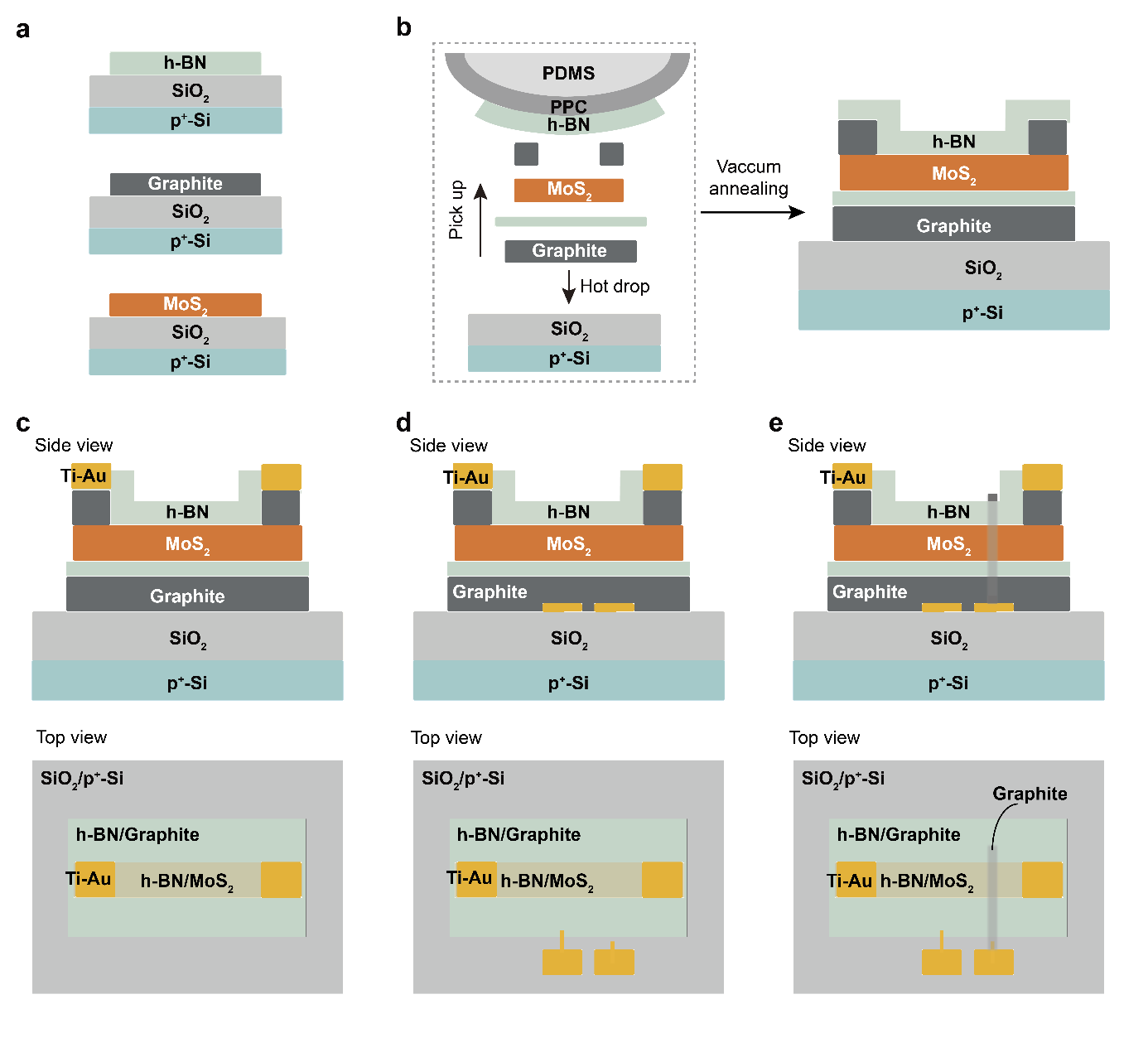
Figure S1.** Illustration of the device fabrication. a) Graphite, MoS_2_, and h-BN were exfoliated from their bulk crystals and were placed on a SiO_2_/p^+^Si substrate. b) h-BN as an encapsulation layer was picked up using a piece of propylene-carbonate (PPC). The graphite electrode, MoS_2_, h-BN and graphite gate were then picked up in sequence. c) The stack was released at 130 °C onto the surface of a 300-nm-thick SiO_2_ layer which was grown on a heavily p or n-doped Si wafer (0.2-0.05 Ω·cm^-1^), followed by heating in a vacuum at 350 °C for 120 min to remove the PPC. d) Metal contacts for source/drain (Ti/Au: 5/50 nm) and the Ti-Au pad (Ti/Au: 1/3 nm) were formed using electron-beam lithography (EBL), reactive ion etching (RIE), electron-beam evaporation and lift-off processes. e) Polydimethylsiloxane (PDMS) was used as the medium to transfer the top graphite gate layer onto the h-BN layer.


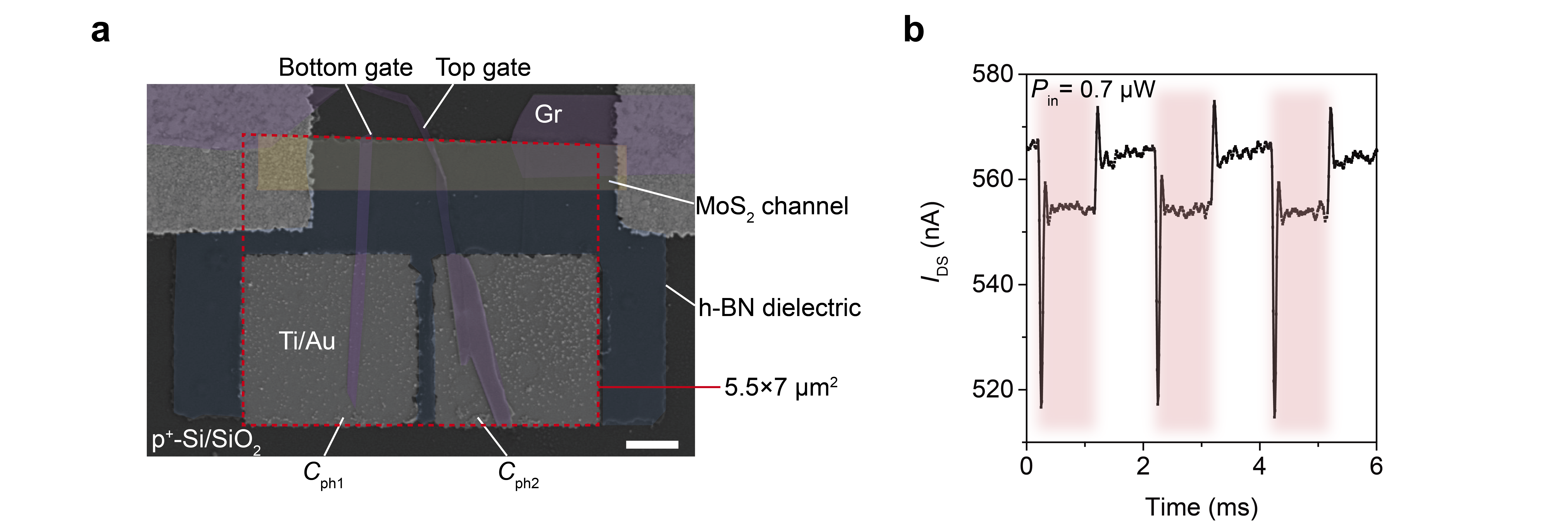


**Figure S2.** False-color scanning electron microscope (SEM) image of the device (scale bar: 1 μm). The charge-coupled phototransistor uses hexagonal boron nitride (h-BN) as the dielectric layer, graphite as the contact electrodes, and molybdenum disulfide (MoS_2_) as the channel. The top and bottom graphite gates are connected to two 3×3 µm^2^ photosensitive capacitors (*C*_ph1,_ *C*_ph2_).

**
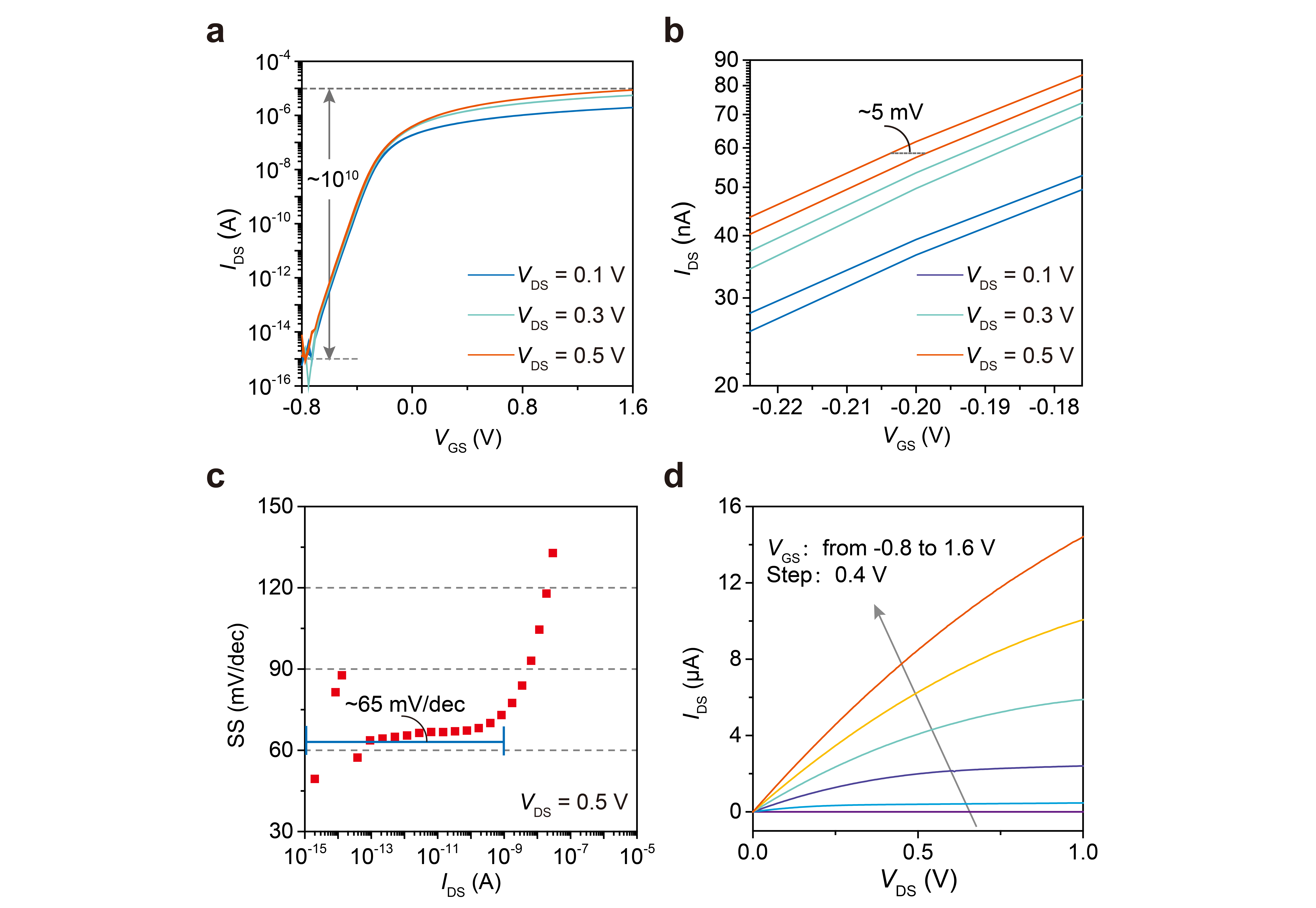
**

**Figure S3.** Electrical performance of the back-gate MoS_2_ transistor with a thick h-BN as the dielectric. a) Transfer characteristic (*I*_DS_-*V*_GS_) curves of the device at different *V*_DS_, showing a large on-to-off current ratio of about 10^10^. b) Transfer characteristics in a smaller bias range indicating a hysteresis of ~5 mV. c) The subthreshold swing **(**SS) can be as low as 65 mV/dec. d) Output characteristics (*I*_DS_-*V*_DS_) of the device at different *V*_GS_ showing that Ohmic contact was made at the source and drain.

**
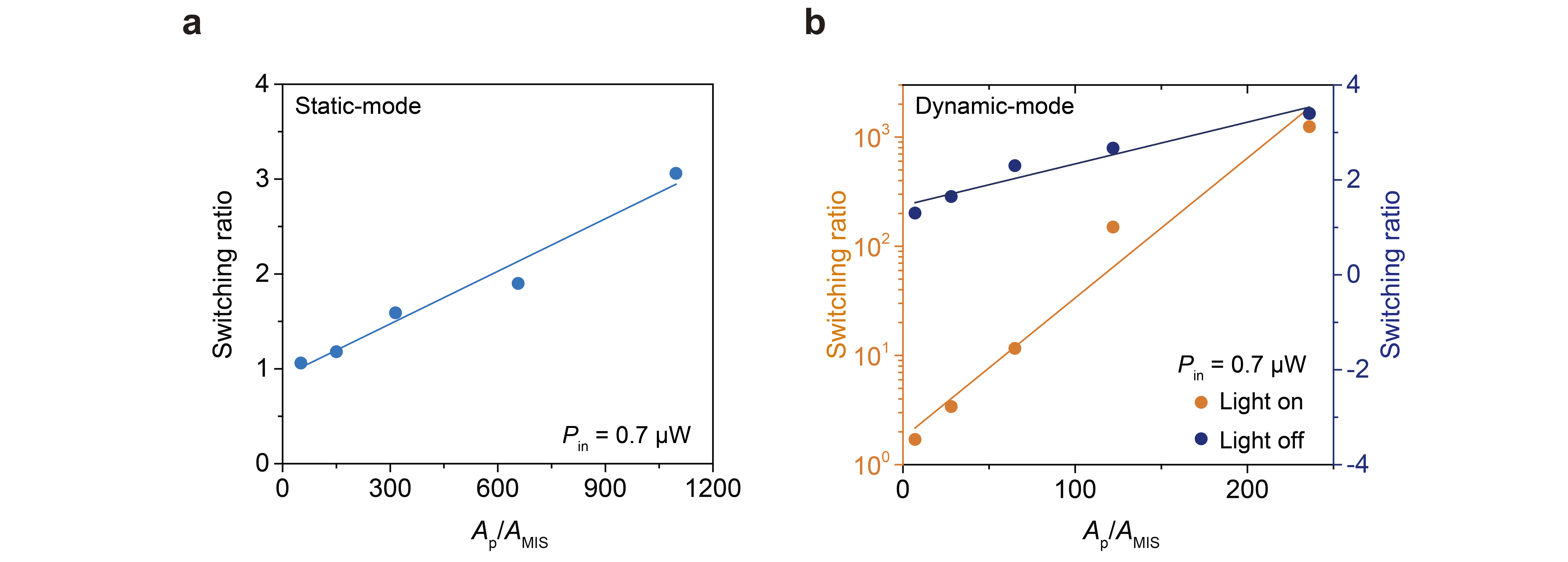
**

**Figure S4.** Switching ratio (*I*_Dark_/*I*_Light_) of the device as the phototransistor (a) or an event-driven pixel (b) increases with the varying ratio of the photosensitive capacitor area to the MIS capacitance area (*A*_p_/*A*_MIS_).

Key factors influencing the performance of the device include the area of the photosensitive capacitors (*A*_p_), the Metal-Insulator-Semiconductor (MIS) capacitance area of the transistors (*A*_MIS_), and the subthreshold swing (SS) of the transistors. Specifically: 1) As the area of the photosensitive capacitors increases, a larger gate voltage will be applied to the channel. On the other hand, when the MIS capacitance area of the transistor decreases, the photogenerated electrons will be distributed over a smaller area, thereby generating a stronger electric field. Therefore, the larger the ratio of the photosensitive capacitor area to the MIS capacitance area (*A*_p_/*A*_MIS_), the more significant the device's photo-response. Consequently, our device can be scaled down by simultaneously reducing the area of the MIS capacitance of the transistor and the photosensitive capacitors while keeping the performances such as the photo-response. 2) A smaller subthreshold swing (SS) of the transistor ensures a more significant current variation under a fixed gate voltage. For example, MoS_2_ transistors (SS = 65 mV/dec) exhibit more sensitive optoelectronic response characteristics compared to carbon nanotube transistors (SS = 180 mV/dec)


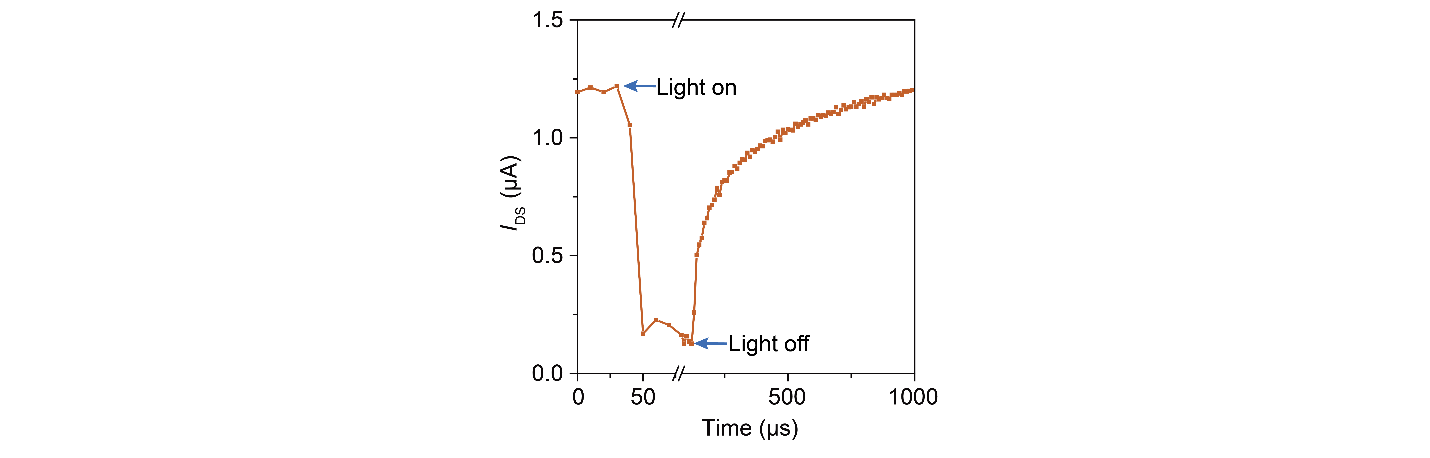


**Figure S5.** Optical switching response of the back-gate MoS_2_ transistor with a thick h-BN as the dielectric, connected with single photosensitive capacitor.


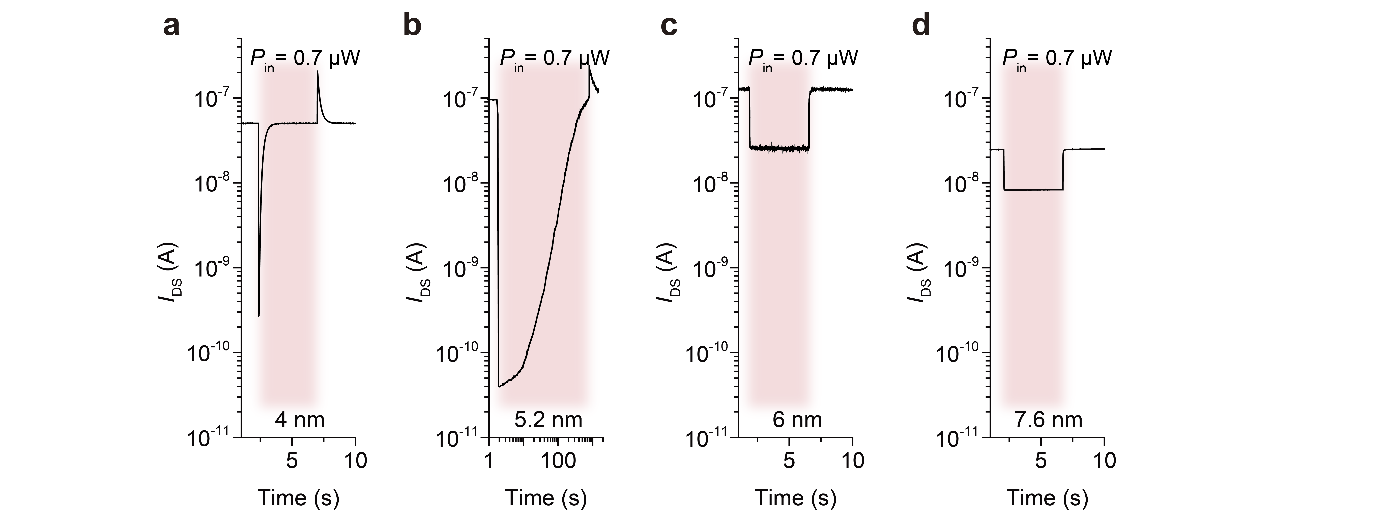


**Figure S6.** The photoelectric behavior of transistors depends on the thickness of h-BN using a 516-nm light with a *P*_in_ of 0.7 μW at *V*_DS_ = 0.1 V. a) 4-nm-thick h-BN. b) 5.2-nm-thick h-BN. c) 6-nm-thick h-BN. d) 7.6-nm-thick h-BN.


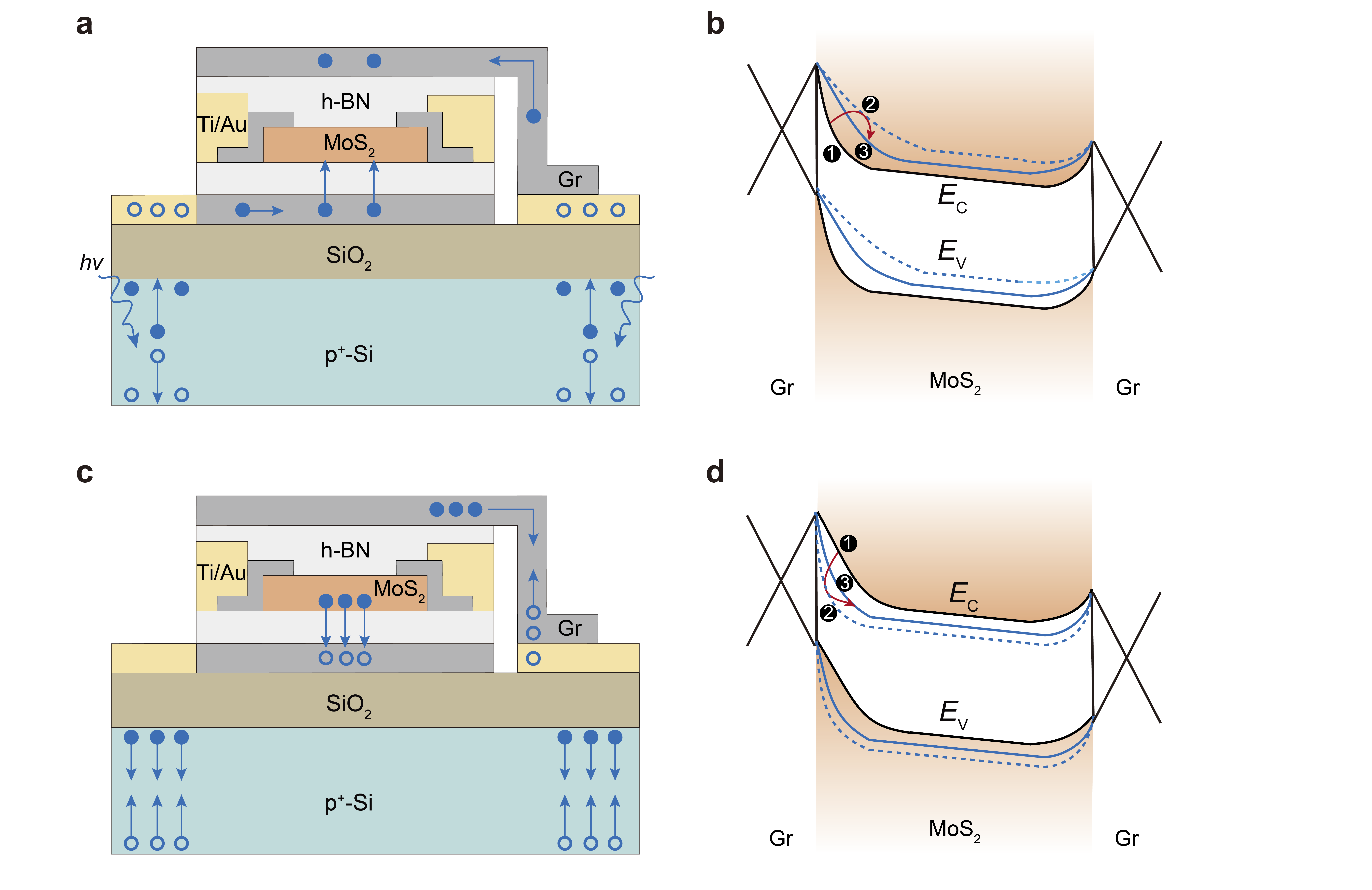


**Figure S7.** Mechanism of the charge-coupled MoS_2_ phototransistor. a) Schematic of the movement of photo-generated carriers under illumination. Gr: graphite. b) Energy band diagrams of the device under illumination. *E*_C_ is the conduction band minimum and *E*_V_ is the valence band maximum. *hv* denotes the incident light; the blue balls and circles indicate negative and positive charges, respectively. The black lines: initial state, the blue dashed line: transient state, and the blue line: stable state. c) Schematic of the movement of photo-generated carriers after removing the light. d) Energy band diagrams of the device after removing the light.

As shown in Figure S7a, the charge-coupled MoS_2_ phototransistor has a thin h-BN dielectric layer above the bottom gate and a thick h-BN dielectric layer below the top gate. In dark, the potential barrier between the graphite source and drain electrodes and the MoS_2_ channel is thin (Figure S7b ①), leading to a relatively large drain current. Under illumination, the photo-generated electrons in the bottom gate tunnel through the thin h-BN layer into the MoS_2_ layer, applying a transient negative gate voltage to the channel, meanwhile, the electrons in the top gate are blocked by the thick h-BN layer and cannot tunnel, thus providing a sustained negative gate voltage for the channel, leading to a thicker potential barrier (Figure S7b ②) and a reduced current. After the tunneling process in the thin h-BN layer completes, only the electrons in the top gate apply a negative gate voltage to the channel (Figure S7b ③). When the illumination is removed, the opposite process occurs in the device (Figure S7cd). This novel mechanism enables the transistor to simultaneously capture dynamic and static information, enhancing its functionality and versatility.


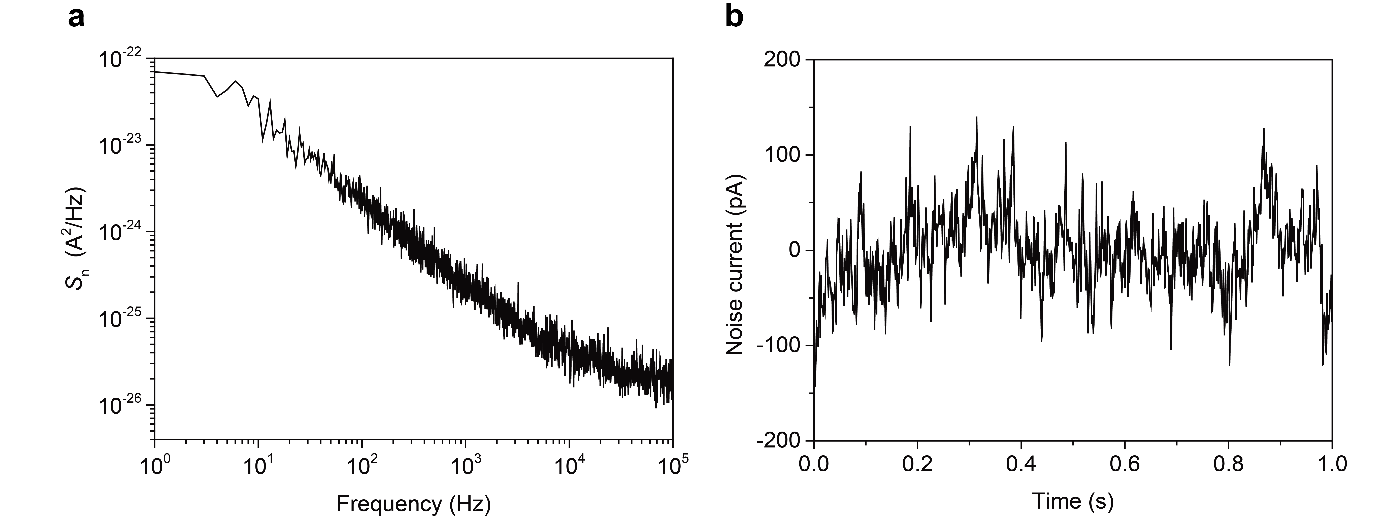


**Figure S8.** Noise characterization of the charge-coupled phototransistor. a) Noise density spectral (*S*_n_) as a function of frequency at *V*_DS_ = 50 mV. b) Noise current extracted from Figure S5a.

Due to the noise current of the device being below 200 pA, it is able to accurately detect photocurrent exceeding 1 nA (Figure 4a).


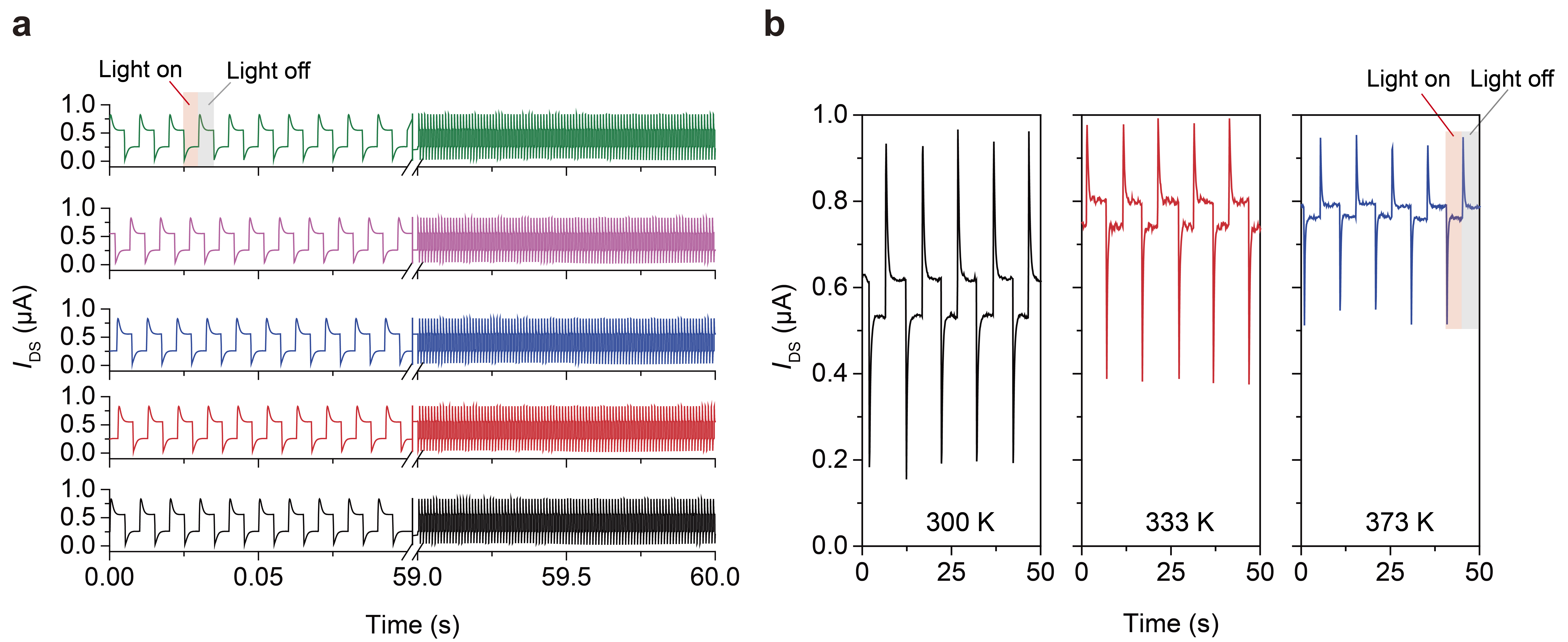


**Figure S9.** Photoresponse of the device at different temperatures, including 300K, 333K, and 373 K, under a 516-nm light with *P*_in_ of 0.7 μW and *V*_DS_ = 0.1 V.


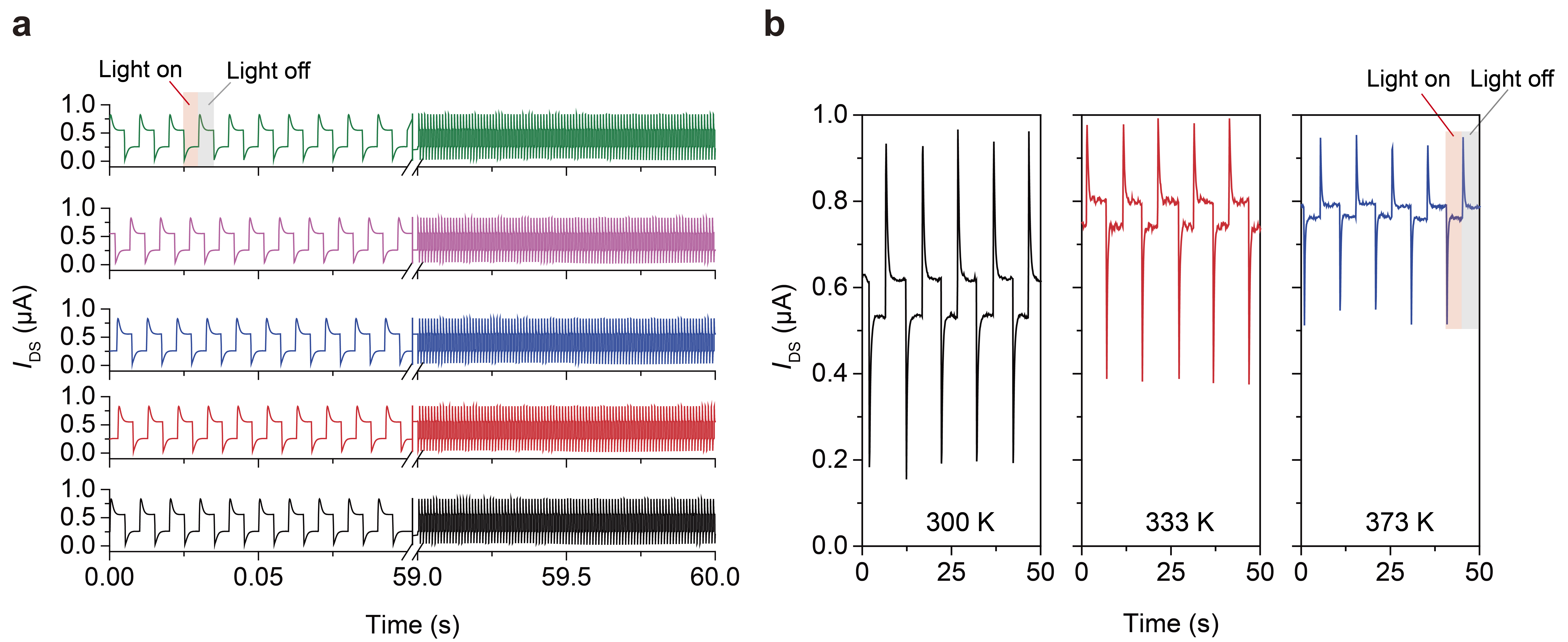


**Figure S10.** Optical switching response of the device under a 516-nm light with *P*_in_ of 0.8 mW and 100 Hz frequency, *V*_DS_ = 0.5 V.

In order to demonstrate the cycle-to-cycle uniformity, we conducted tests for 30,000 cycles (6,000 cycles per time, five times) of the photoelectric response of the device, showing excellent cycle-to-cycle uniformity (Figure S10).


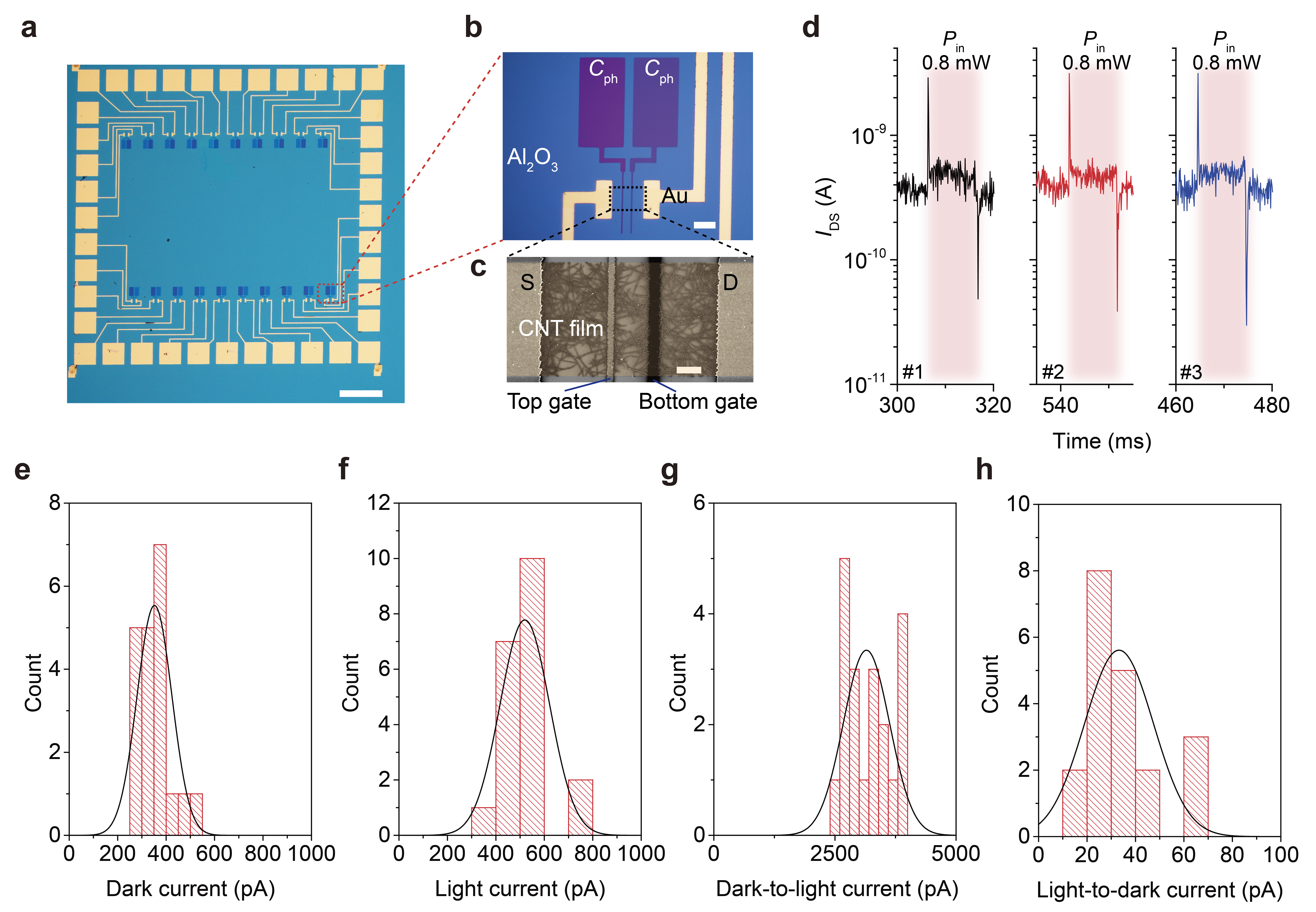


**Figure S11.** CNT-based charge-coupled phototransistors. a) Optical image of the charge-coupled phototransistor array. Scale bar: 200 μm. b) Optical image of a charge-coupled phototransistor. Scale bar: 10 μm. c) Scanning electron microscope (SEM) image of the channel of this device. Scale bar: 2 μm. d) Photoresponse behavior of three devices under a 516-nm light with *P*_in_ of 0.8 mW and *V*_DS_ = 1 V. e-h) Histograms of device currents including dark current (e), light current (f), dark-to-light current (g), and light-to-dark current (h).

Our device concept can be realized using various materials. To demonstrate device-to-device uniformity, we used a large area carbon nanotube (CNT) film instead of mechanically exfoliated 2D materials to fabricate the device, where CNT was used as the channel material, aluminum oxide (Al_2_O_3_) as both the bottom and top dielectric layers and gold as contact electrodes. 20 CNT-based charge-coupled phototransistors were fabricated on the same wafer (Figure S11a-c). Because that the CNT was a p-type semiconductor, the devices exhibited an opposite light response behavior compared to the n-type MoS_2_ devices, where a positive current spike appeared under a light illumination, while a negative current spike appeared once the light was removed (Figure S11d). By statistically analyzing the dark and light currents in the static response and the dark-to-light and light-to-dark currents in the transient response of these 20 devices, we observed excellent device uniformity (Figure S11e-h).


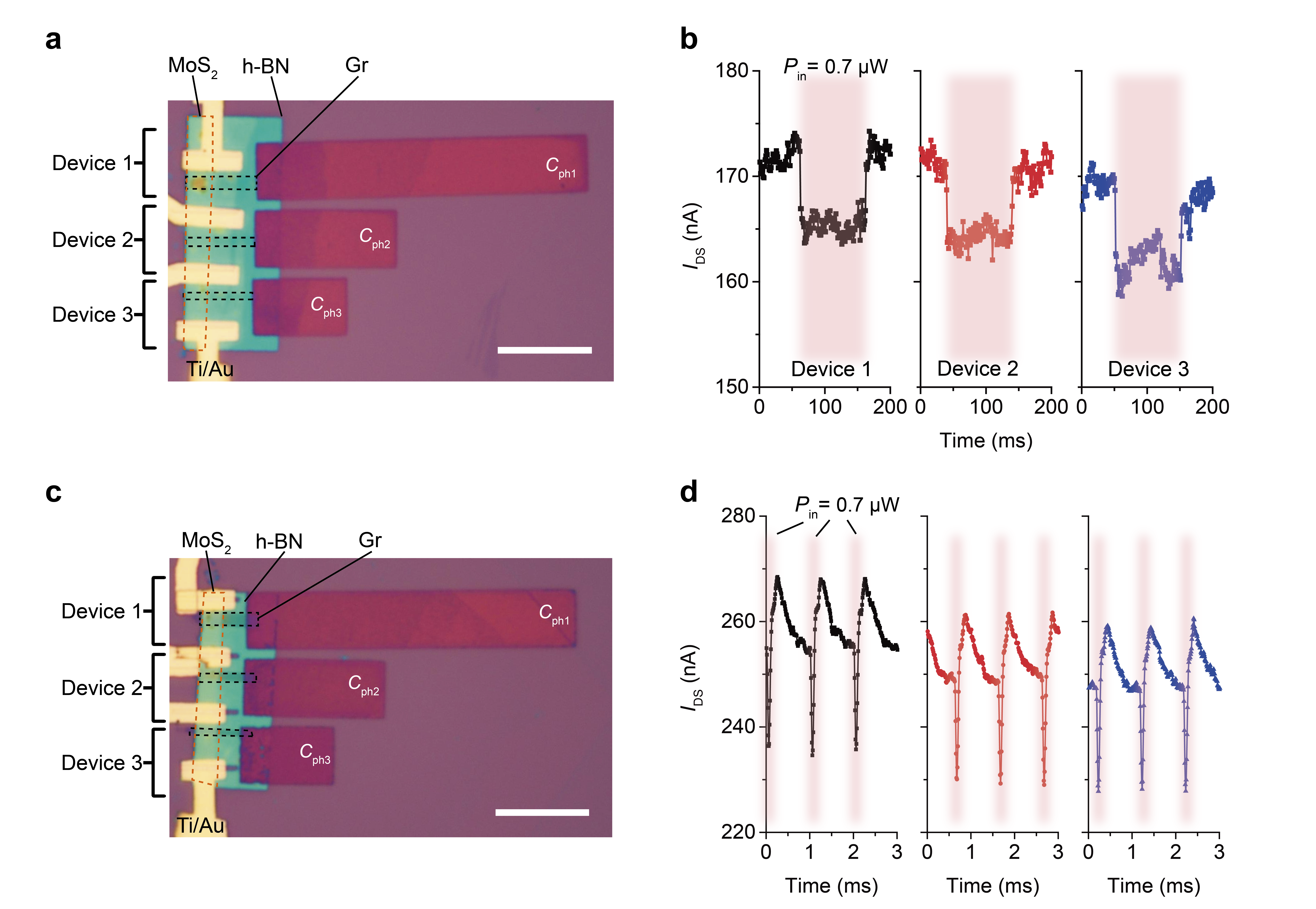


**Figure S12.** Three devices fabricated using the same MoS_2_ channel, h-BN dielectric layer, and Ti-Au electrodes, with varying *A*_p_ and *A*_MIS_ but a consistent *A*_p_/*A*_MIS_ ratio of 30. (a) Devices utilizing a thick h-BN dielectric, functioning as phototransistors (b). (c) Devices employing a thin h-BN dielectric, operating as event-driven pixels (d).

We fabricated three transistors using the same MoS_2_ channel, h-BN dielectric layer, and Ti-Au electrodes, with different *A*_p_ and *A*_MIS_ but the same *A*_p_/*A*_MIS_ ratio (Fig. S12a, c). Regardless of whether they were used for static or dynamic detection, these devices exhibited similar photoresponse characteristics (Fig. S12b, d). Thus, by simultaneously reducing both the *A*_p_ and *A*_MIS_ of the transistor, our device can be further miniaturized while maintaining excellent performance.


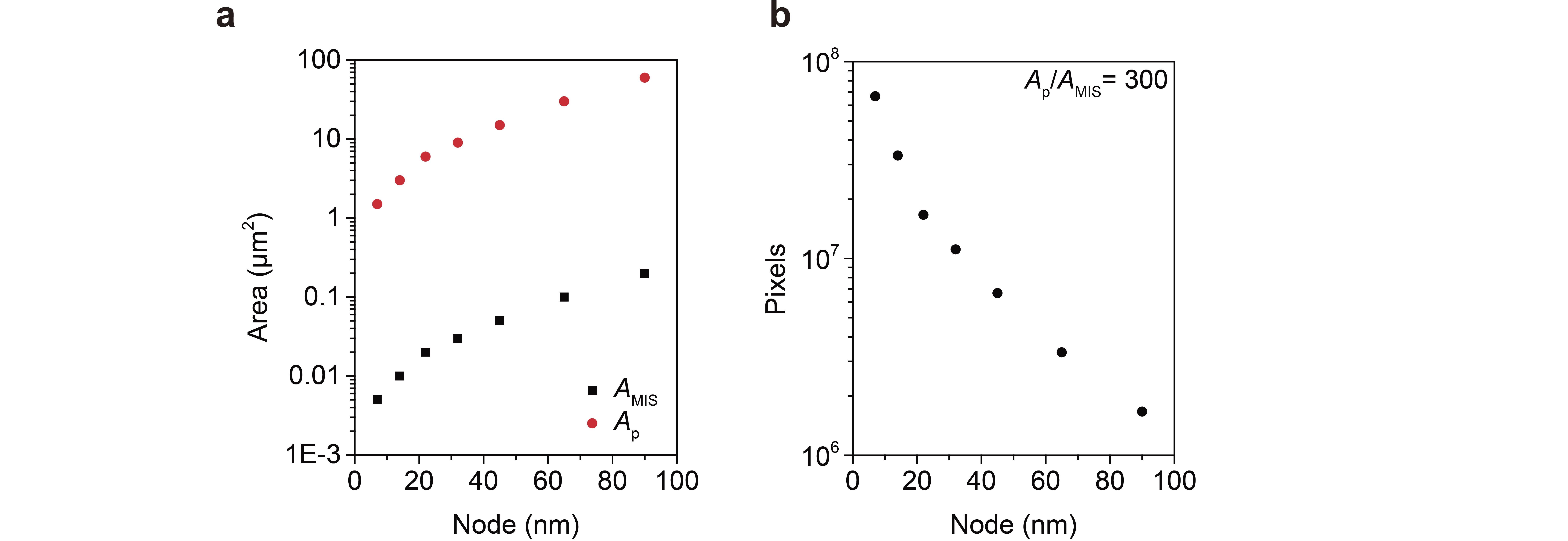


**Figure S13.** Future application prospects of charge-coupled phototransistors. (a) MIS capacitance area and photosensitive capacitor area corresponding to different silicon process nodes, with a ratio of 1:300. (b) Integration density of charge-coupled phototransistors at different silicon process nodes when the ratio of photosensitive capacitor area to MIS capacitance area is 300.

Based on the above experimental results, it can be concluded that for the materials currently used, it is highly feasible to further reduce the device size while maintaining sensitivity by simultaneously shrinking both the area of the photosensitive capacitors and the MIS capacitance area of the transistor, while keeping their ratio unchanged. Additionally, the principle of our device demonstrates strong versatility. For mature silicon-based technologies (*IEEE. International Roadmap for Devices and Systems (IRDS)*), in the 32 nm process node, the subthreshold swing (SS) of silicon MOSFETs is typically below 80 mV/dec, and the MIS capacitance area can be as low as 0.01 - 0.03 μm^2^ (Fig. S13a). Under these conditions, even with a photosensitive capacitor to MIS capacitance area ratio of 300:1, it is still possible to achieve integration densities exceeding tens of millions of pixels on a 1×1 cm substrate (Fig. S13b).


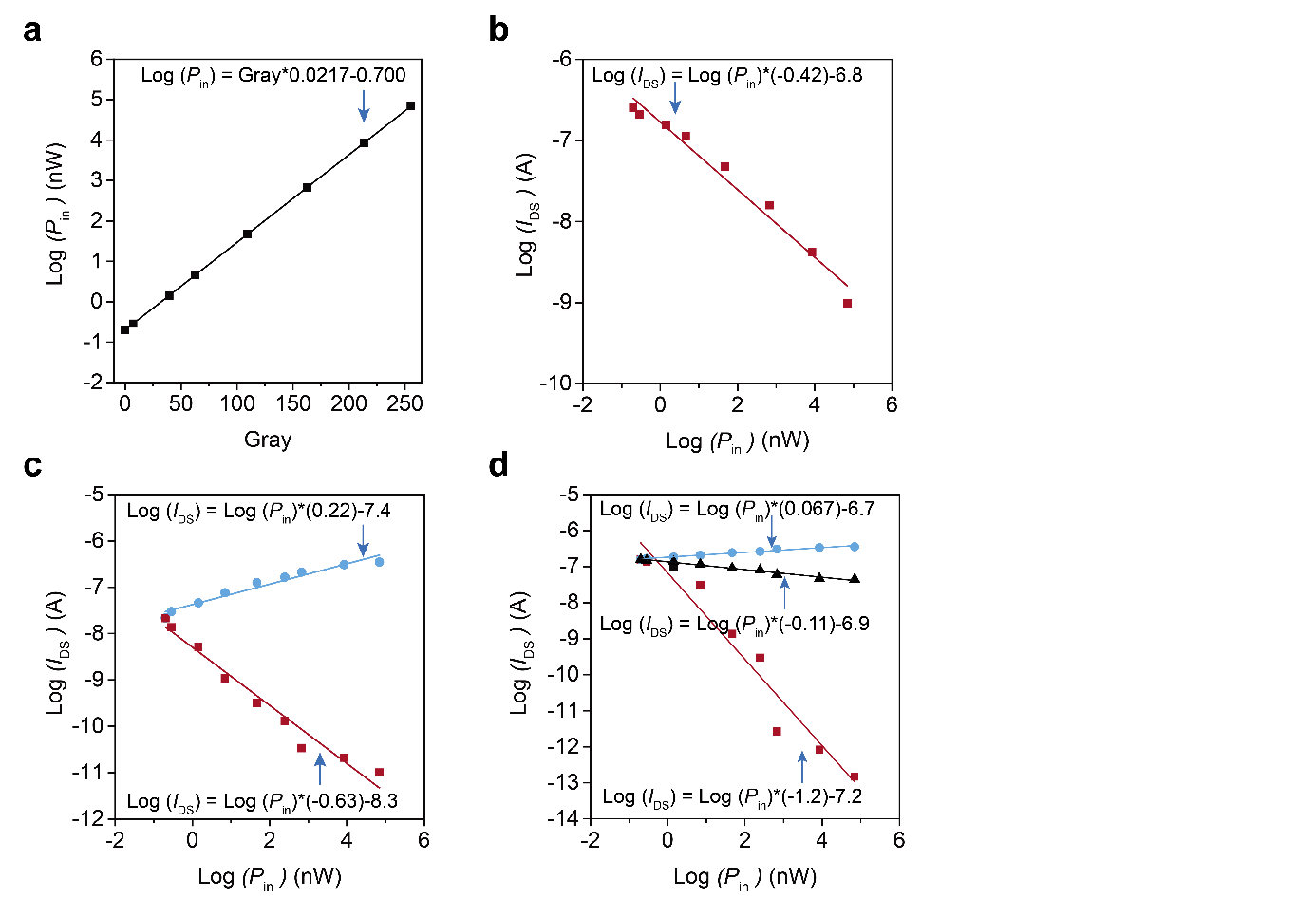


**Figure S14.** Image sensor simulations based on three-mode sensors. a) Conversion between gray values and light power. b-d) Current (*I*_DS_) as a function of light power (*P*_in_). b) Frame-based sensor, c) Event-based sensor (dark-to-light current, red line; light-to-dark current, blue line), d) Charge-coupled phototransistor (dark-to-light current, red line; light-to-dark current, blue line; light-on current, black line).


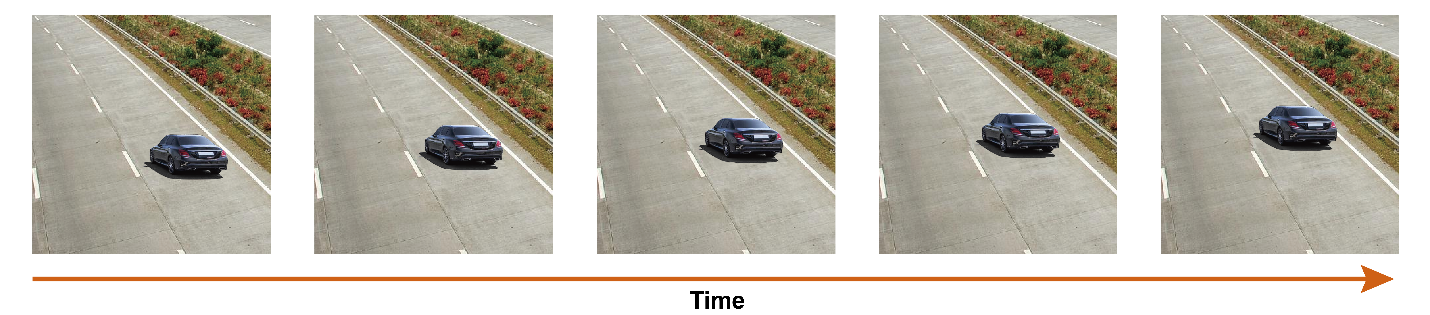
**Figure S15.** A self-designed scene containing a moving car and static roads. The car travels along the road from the bottom right corner to the top left corner.

**Table S1.** Benchmarks of the charge-coupled phototransistor against traditional event-based sensors.


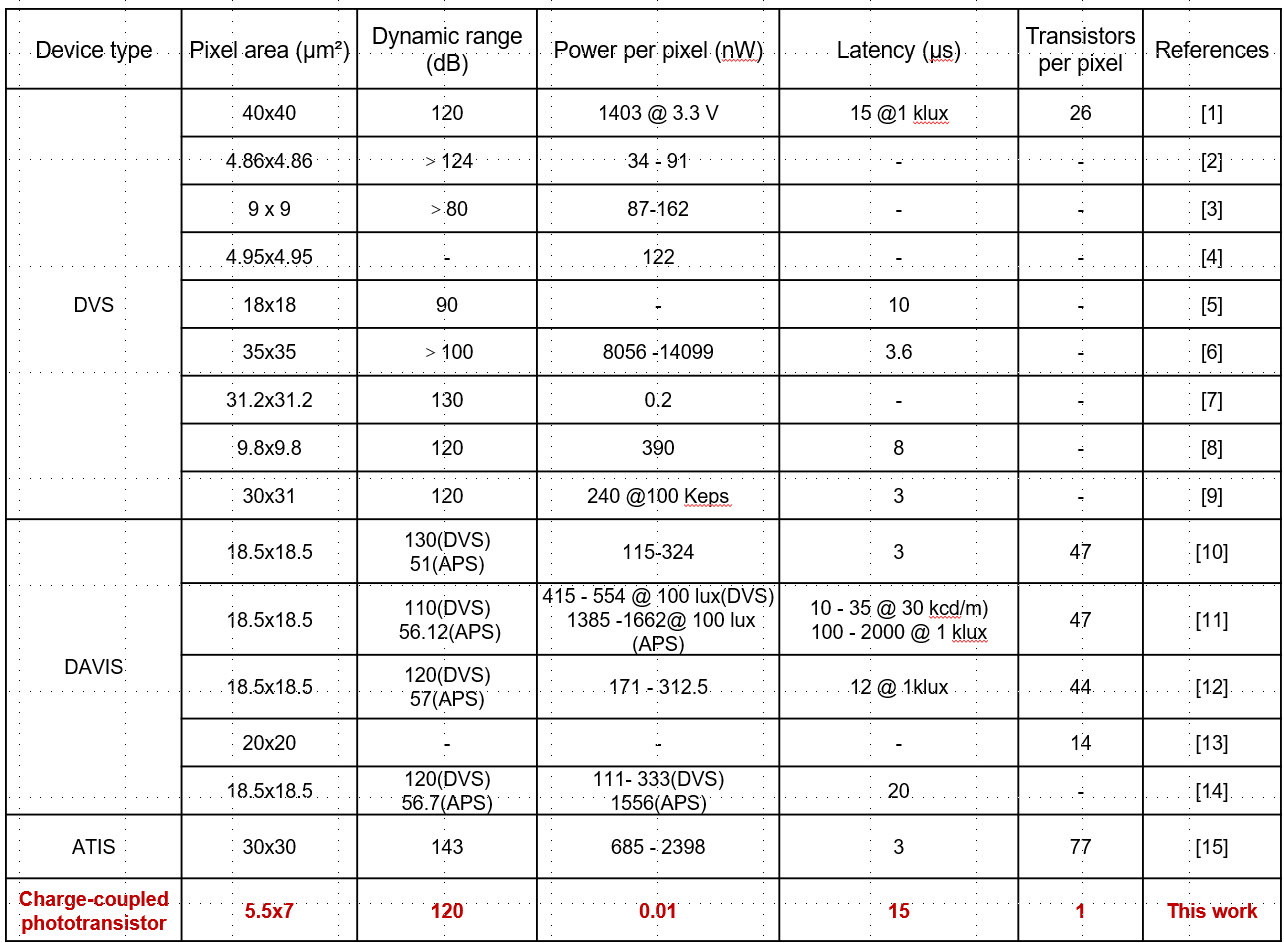


In order to better highlight the performance advantages of our device, we have conducted a comprehensive comparison with existing devices in relevant categories, such as dynamic vision and active pixel sensors (DAVIS), dynamic vision sensors (DVS), and asynchronous time based image sensors (ATIS), as summarized in Table S1. The comparison includes key parameters such as size, dynamic range, power consumption, latency, and the number of transistors in the pixel unit. Our analysis reveals several significant advantages of our device. Firstly, it utilizes the fewest transistors, which significantly reduces both complexity and power consumption. Secondly, compared to existing DAVIS devices, our device’s size is reduced by 8 to 10 times, showing the strong potential for enhanced system integration and spatial efficiency. Thirdly, our device demonstrates excellent dynamic range and lower power consumption, making it more promising for high-density integration and low-power applications.

**References**

1. P. Lichtsteiner, C. Posch, T. Delbruck, *IEEE J. Solid-State Circuit.* **2008**, *43*, 566.
2. T. Finateu, A. Niwa, D. Matolin, K. Tsuchimoto, A. Mascheroni, E. Reynaud, in *2020 IEEE International Solid-State Circuits Conference - (ISSCC)*, San Francisco, CA, USA, **2020**, pp. 112-114.
3. B. Son, Y. Suh, S. Kim, H. Jung, J. -S. Kim, C. Shin, K. Park, K. Lee, J. Park, J. Woo, Y. Roh, H. Lee, Y. Wang, I. Ovsiannikov, H. Ryu, in 2017 *IEEE International Solid-State Circuits Conference (ISSCC)*, San Francisco, CA, USA, **2017**, pp. 66-67.
4. Y. Suh, S. Choi, M. Ito, J. Kim, Y. Lee, J. Seo, H. Jung, D. -H. Yeo, S. Namgung, J. Bong, S. Yoo, S. -H. Shin, D. Kwon, P. Kang, S. Kim, H. Na, K. Hwang, C. Shin, J. -S. Kim, P. K. J. Park, J. Kim, H. Ryu, Y. Park, in 2020 *IEEE International Symposium on Circuits and Systems (ISCAS)*, Seville, Spain, **2020**, pp. 1-5.
5. M. Guo, J. Huang, S. Chen, in 2017 *IEEE International Symposium on Circuits and Systems (ISCAS),* Baltimore, MD, USA, **2017**, pp. 1-1.
6. J. A. Lenero-Bardallo, T. Serrano-Gotarredona, B. Linares-Barranco, *IEEE J. Solid-State Circuit.* **2011**, *46*, 1443.
7. M. Yang, S.C. Liu, T. Delbruck, *IEEE J. Solid-State Circuits* **2015**, *50*, 2149.
8. S. Chen, M. Guo, in 2019 *IEEE/CVF Conference on Computer Vision and Pattern Recognition Workshops (CVPRW),* Long Beach, CA, USA, **2019**, pp. 1682-1683.
9. T. Serrano-Gotarredona, B. Linares-Barranco, *IEEE J. Solid-State Circuit.* **2013**, *48*, 827.
10. C. Brandli, R. Berner, M. Yang, S. C. Liu, T. Delbruck, *IEEE J. Solid-State Circuit.* **2014**, *49*, 2333.
11. D. P. Moeys, F. Corradi, C. H. Li, S. A. Bamford, L. Longinotti, F. F. Voigh, *IEEE Trans. Biomed. Circuits Syst.* **2018,** *12*, 123.
12. R. Berner, C. Brandli, M. Yang, S. C. Liu, T. Delbruck, in 2013 *Symposium on VLSI Circuits,* Kyoto, Japan, **2013**, pp. C186-C187.
13. C. H. Li, C. Brandli, R. Berner, H. J. Liu, M. H. Yang, S. C. Liu, in 2015 *IEEE International Symposium on Circuits and Systems (ISCAS),* Lisbon, Portugal, **2015**, pp. 718-721.
14. iniVation. DAVIS 346, https://inivation.com/wp-content/uploads/2019/08/DAVIS346.pdf accessed: November, 2024.
15. C. Posch, D. Matolin, R. Wohlgenannt, *IEEE J. Solid-State Circuit.* **2011**, *46*, 259.
